# Characteristics of US Adults Delaying Dental Care Due to the COVID-19 Pandemic

**DOI:** 10.1177/2380084420962778

**Published:** 2021-01

**Authors:** A.M. Kranz, G. Gahlon, A.W. Dick, B.D. Stein

**Affiliations:** 1 RAND Corporation, Arlington, VA, USA; 2 RAND Corporation, Boston, MA, USA; 3 RAND Corporation, Pittsburgh, PA, USA

**Keywords:** dental care delivery, health services research, coronavirus, health services accessibility, health care surveys, cross sectional analysis

## Abstract

**Background:**

Coronavirus disease 2019 (COVID-19) has disrupted the delivery of health care services, including dental care. The objective of this study was to quantify and describe US adults who delayed dental care due to the COVID-19 pandemic.

**Methods:**

We analyzed cross-sectional responses collected from a nationally representative and long-running panel survey of US adults conducted in late May and early June 2020 (response rate = 70%). The survey included questions about dental care delayed due to the COVID-19 pandemic, purpose of the delayed dental visits, timing of future dental visits, and demographic information. Pearson’s chi-square tests were used to determine if rates of delayed dental care varied by subgroup. A multivariable regression model, adjusted for age, race, Hispanic ethnicity, census division, and rurality, was estimated to predict the odds of reporting delayed dental care.

**Results:**

Nearly half of respondents (46.7%) reported delaying going to the dentist or receiving dental care due to the COVID-19 pandemic. Among adults who reported delaying dental care due to the pandemic, 74.7% reported delaying a checkup, 12.4% reported delaying care to address something that was bothering them, and 10.5% reported delaying care to get planned treatment. About 44.4% of adults reported that they planned to visit the dentist within the next 3 mo. In the multivariable regression model, only living in an urban (vs. rural) area was associated with significantly higher odds of delayed dental care due to the pandemic (odds ratio: 1.5; 95% confidence interval: 1.1, 2.1).

**Conclusions:**

Nearly half of US adults reported delaying dental care due to the COVID-19 pandemic during the spring of 2020. Our results offer insight into the experiences of patients seeking dental care this spring and the economic challenges faced by dental providers due to the pandemic.

**Knowledge Transfer Statement:**

This article describes US adults who delayed dental care due to the COVID-19 pandemic. Results can be used by clinicians and policymakers to understand delayed care during the pandemic.

## Introduction

Coronavirus disease 2019 (COVID-19) has disrupted all facets of daily life, which includes the delivery of health care services. Studies have already documented declines in use of health care services due to the COVID-19 pandemic (Gelburd 2020; Hartnett et al. 2020), including a 37% decline in outpatient medical visits in the United States from March 15 to June 20, 2020 (Mehrotra et al. 2020). However, less is known about use of dental care during the COVID-19 pandemic.

Common dental procedures generate aerosols and droplets, leading to risk of COVID-19 transmission in dental practices (Izzetti et al. 2020; Meng et al. 2020). Given the risk of virus transmission, many dental offices throughout the world closed or offered only urgent care during the spring of 2020. A survey of 650 international dental care professionals in 30 countries during mid-March 2020 found that more than two-thirds of respondents wanted to keep their dental practices closed until virus cases declined and 87% indicated that they were afraid of being infected from a patient or coworker (Ahmed et al. 2020). In the United States, many states encouraged or required dental offices to deliver only emergency care for most of March and April 2020 to decrease the risk of virus transmission. During the week of April 6, 2020, a survey by the American Dental Association (ADA) Health Policy Institute (2020) found that 97.1% of dentists reported that their offices were seeing emergency patients only or not seeing any patients. As cases declined in the United States and the ADA and US Centers for Disease Control and Prevention (CDC) issued guidance for dental care (ADA 2020c; CDC 2020b), dental offices began to reopen, with 64.7% and 90.1% of dentists reporting their offices being open as of May 18 and June 1, 2020, respectively. While these surveys provide a snapshot about availability of dental care in the United States, the generalizability is uncertain because the weekly surveys generally report a response rate of less than 10%. Furthermore, these surveys do not provide information on the types of patients who may not be receiving care, which is an important issue given that the COVID-19 pandemic has disproportionally impacted Black and Hispanic individuals (CDC 2020a) and individuals residing in urban areas in the United States during the spring of 2020 (Frey 2020). We are unaware of studies examining delays in dental care and if delays in dental care differed from that experienced by White individuals and individuals residing in rural areas.

Using responses from a nationally representative survey of adults in the United States, conducted in late May and early June, this study quantifies the percentage of adults who delayed dental care due to the COVID-19 pandemic, explores the purpose of the delayed dental visits, and describes when adults plan to visit dental offices. In addition, we describe adults who reported delaying dental care due to the pandemic by location and across a range of demographic characteristics.

## Methods

This study used the RAND American Life Panel (ALP) survey, a nationally representative Internet panel. Respondents were initially recruited through random-digit dialing. Panel members are invited to participate in online surveys once or twice per month on average and compensated financially for each survey to increase response rates and representativeness. Demographic information is collected on panelists 3 times a year, most recently in February 2020. Additional information on the technical aspects of the ALP, including weighting methodology, is available in full detail elsewhere (Pollard and Baird 2017). In addition to questions about dental care delayed due to the pandemic, the survey included questions about voting behavior and mental health care. For this survey, 3,402 panel members were invited to participate in the survey. The survey was in the field from May 18 to June 8, 2020, and achieved a response rate of 70.2% (*N* = 2,387).

Panelists were asked, “Due to the COVID-19 pandemic, have you delayed or forgone getting dental care or going to the dentist?” Five response options were available: yes; no, I still went to the dental office; no, I had a virtual appointment via the Internet, phone, or video; no, I wasn’t planning to go to the dentist; or other. Panelists responding with “other” were able to write in a reason. Panelists who indicated they had delayed dental care due to the pandemic were asked, “What was the primary reason for the dental visit that was delayed or foregone?” Response options included get a check-up, examination, or cleaning; something was wrong, bothering, or hurting me; to get treatment of a condition that the dentist discovered at an earlier checkup or examination; or don’t know. These response options were similar to options used in the National Health and Nutrition Examination Survey when asking about the main reason that the respondent last visited a dentist (CDC 2020c). All panelists were asked, “When do you plan to visit the dentist?” Response options included this month, within next 3 mo, within next 6 mo, within next 12 mo, more than 1 y from now, or never.

Demographic information included age in years (categorized into 4 groups:<40 y, 40–54 y, 55–64 y, or 65 y and older), race (White, Black or African American, American Indian or Alaskan Native, Asian or Pacific Islander, or other), and a dichotomous indicator of Hispanic ethnicity. Geographic information included a 9-level variable identifying the US census division and a dichotomous indicator of living in a small to midsize city or large city, with a population of 50,000 or more people (referred to as “urban”), versus living in a rural or small town, with a population fewer than 50,000 people (referred to as “rural”).

Univariate statistics were generated to describe panelists’ responses to each item related to dental care. Bivariate statistics were generated to describe the percentage of adults who reported delaying dental care due to the pandemic for each US census division, rurality, and a range of demographic characteristics (i.e., age, race, Hispanic ethnicity). Pearson’s chi-square tests were conducted to assess if rates of delayed dental care varied by subgroup. A multivariable regression model predicting the odds of reporting delayed dental care (vs. not reporting delayed dental care) was estimated and adjusted for age, race, Hispanic ethnicity, census division, and rurality. We used complete case analysis, analyzing responses for which there were no missing data on the variables of interest. All analyses incorporated ALP survey weighting. This study was determined to be exempt by our organization’s institutional review board and conforms to the STROBE guidelines.

## Results

**Figure 1.**
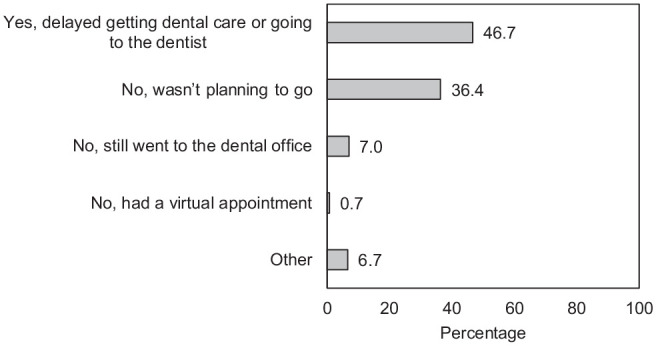
Percentage of adults reporting delayed use of dental care due to the COVID-19 pandemic.

Survey respondents were predominantly White (75.1%), were not Hispanic (81.1%), and lived in urban areas (78.5%), as described in the Appendix Table. Nearly half of US adults (46.7%) reported delaying going to the dentist or receiving dental care due to the COVID-19 pandemic (Fig. 1). Fewer than 1 in 10 adults reported that they still went to the dental office (9.3%), and 0.7% indicated that they had a virtual dental appointment.

**Figure 2.**
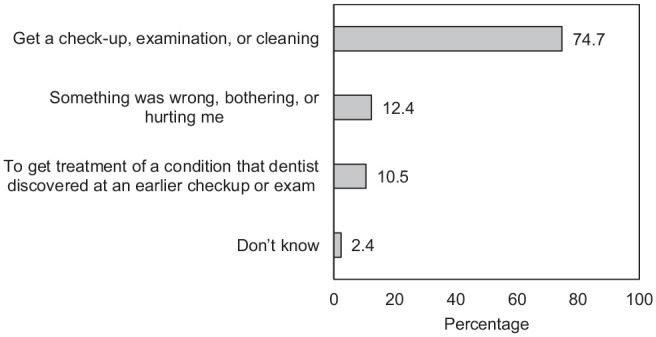
Primary reason for the dental visit that was delayed among adults who reported delaying dental care due to the COVID-19 pandemic (%).

Among adults who reported delaying dental care due to the COVID-19 pandemic, nearly 3 in 4 adults (74.7%) reported delaying care for a checkup, examination, or cleaning (Fig. 2). Fewer adults reported delaying care to address something that was wrong, bothering, or hurting them (12.4%) or to get treatment of a condition that a dentist discovered during an earlier visit (10.5%).

**Figure 3.**
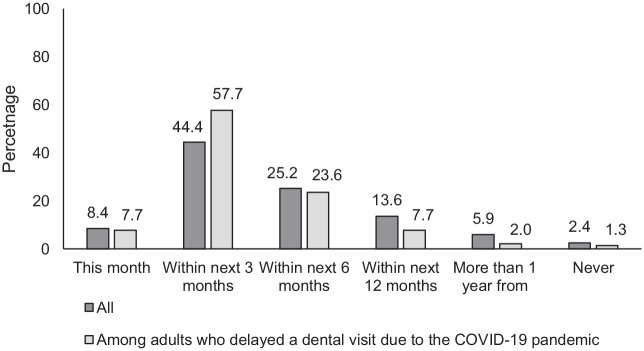
Percentage of adults reporting when they plan to visit the dentist, overall and among adults who reported delaying dental care due to the COVID-19 pandemic.

Only 8.4% of adults reported that they plan to visit the dentist this month (Fig. 3). Among those who had delayed care and were planning a dental visit during the next month, 57.6% had delayed a checkup and 26.2% had delayed care for treatment. About 44.4% of adults reported that they planned to visit the dentist within the next 3 mo, and this rate was even higher, at 57.7%, among adults who reported delaying dental care due to the COVID-19 pandemic.

Few differences in reported delays of dental care during our survey period were observed across a range of characteristics (Table 1). Adults in rural areas (38.0%) were significantly less likely to report delayed dental care due to the COVID-19 pandemic than adults in urban areas (49.1%, *P* = 0.008). No other significant differences were observed across subgroups. Women were slightly more likely to report delaying dental care than men (49.5% vs. 43.7%, *P* = 0.13). More than half of Black adults (51.7%) reported delaying dental care, which was slightly higher than the percentage of White adults reported delaying dental care (45.2%, *P* = 0.40). Hispanic (47.0%) and non-Hispanic (46.6%) adults reported similar rates of delayed dental care (*P* = 0.94). Reports of delayed dental care did not significantly vary by census division (*P* = 0.43). Adults living in the East South Central census division (Alabama, Kentucky, Mississippi, and Tennessee) had the lowest rate of reported delayed dental care (34.0%) and adults in the New England census division (Connecticut, Maine, Massachusetts, New Hampshire, Rhode Island, Vermont) had the highest rate of reported delayed dental care (55.2%).

**Table 1.** Percentage of US Adults Reporting Delayed Use of Dental Care Due to the COVID-19 Pandemic by Demographic Factors.

Characteristic	Weighted Row Percentage	Unweighted Count
All	46.7	2,387
Sex		
Male	43.7	1,024
Female	49.5	1,363
Age group		
<40 y	47.9	286
40–54 y	48.2	566
55–64 y	47.0	665
65 y and older	43.2	871
Race		
White	45.2	1,920
Black	51.7	221
American Indian and Alaskan Native	26.6	27
Asian and Pacific Islander	66.6	71
Other	49.9	148
Hispanic ethnicity		
Hispanic	47.0	334
Not Hispanic	46.6	2,054
Rurality^a^		
Urban	49.1	1,860
Rural	38.0	521
Census division of current residence^b^		
New England	55.2	111
Middle Atlantic	48.0	321
East North Central	48.1	312
West North Central	51.6	123
South Atlantic	46.5	399
East South Central	34.0	93
West South Central	40.9	343
Mountain	41.2	258
Pacific	52.1	426

Weighted percentages.

a Six respondents had missing information about rurality.

b One respondent had missing information about census division.

In a multivariable regression model predicting the odds of reporting delayed dental care due to the COVID-19 pandemic (Table 2), only living in an urban area (vs. rural) was associated with significantly higher odds of delayed dental care (odds ratio: 1.5; 95% confidence interval [CI]: 1.1, 2.1; *P* = 0.015). Women were 1.3 times more likely to report delayed dental care (95% CI: 0.9, 1.8; *P* = 0.06).

**Table 2.** Odds of Delaying Dental Care Due to COVID-19 Pandemic among US Adults (*N* = 2,380).

Characteristic	Odds Ratio	95% Confidence Interval
Female (reference group: male)	1.31*	0.98, 1.76
Age group (reference group: <40 y)		
40–54 y	1.04	0.67, 1.62
55–64 y	1.03	0.66, 1.61
65 y and older	0.90	0.59, 1.38
Race (reference group: White)		
Black/African American	1.21	0.68, 2.13
American Indian or Alaskan Native	0.38	0.11, 1.32
Asian or Pacific Islander	2.17	1.00, 4.72
Other	1.08	0.62, 1.88
Hispanic ethnicity (reference group: not Hispanic)	1.14	0.76, 1.72
Urban (reference group: rural)	1.50**	1.08, 2.08
US census division (reference group: New England)		
Middle Atlantic	0.66	0.28, 1.54
East North Central	0.80	0.34, 1.85
West North Central	0.91	0.38, 2.21
South Atlantic	0.67	0.29, 1.52
East South Central	0.46	0.18, 1.18
West South Central	0.54	0.24, 1.23
Mountain	0.60	0.26, 1.39
Pacific	0.75	0.33, 1.69

Results from multivariable logistic regression model predicting the odds of delaying dental care due to COVID-19 pandemic among a US nationally representative sample of 2,380 adults. Six respondents had missing information about rurality and 1 respondent had missing information about census division.

* *P* < 0.10. **P < 0.05.

## Discussion

This nationally representative survey of US adults found that nearly half of adults surveyed during late May to early June 2020 reported delaying going to the dentist or receiving dental care due to the COVID-19 pandemic. Nearly 3 in 4 adults (74.7%) reported delaying care for a checkup, examination, or cleaning, suggesting that most adults who delayed care were unlikely to have had an urgent need, be in pain, or have experienced worse oral health–related quality of life due to delayed care. Although it was not the majority of respondents, 12.4% of adults who reported delaying dental care indicated that the delayed visit was to address something that was wrong, bothering, or hurting them. We do not know if respondents sought care elsewhere, such as from an emergency department (ED). Although EDs have a history of being used by patients to address dental pain and nontraumatic dental conditions (Wall and Vujicic 2015), it is likely that many adults avoided ED visits for dental concerns during this time. Visits for emergency dental services declined by nearly 40% in Beijing from January 1 to February 10, 2020 (Guo et al. 2020), and ED visits in 47 US states declined 42% during the pandemic compared to the prior year (Hartnett et al. 2020).

We found that rates of adults who reported delaying dental care were similar across demographic characteristics, differing only for adults living in rural and urban areas. In the United States, during the time when this survey was fielded, cases of COVID-19 were more common in urban areas (Frey 2020), which aligns with our finding that living in urban areas was associated with significantly higher odds of delayed dental care. However, as the pandemic has spread in the United States, there is growing concern about the disease burden and health care system demands faced outside of urban population centers (Miller et al. 2020), particularly for Black Americans (Sood and Sood 2020). In addition, we failed to find evidence that reports of delaying dental care differed by race and ethnicity. Racial and ethnic inequities in use of dental care prepandemic are well documented, with non-White adults being less likely to visit a dentist (Zhang 2016), yet our analyses suggest that non-White adults were not disproportionally affected as it relates to delayed dental care. Because the COVID-19 pandemic has disproportionally impacted Black and Hispanic individuals (CDC 2020b) and because Black and Hispanic adults are more likely to have unmet dental needs than White adults (Bhoopathi et al. 2020), it is important to monitor and confirm access to dental care among non-White populations during the pandemic.

For dental providers, our results reinforce the economic challenges of the pandemic. Our results that nearly half of adults delayed dental care due to the pandemic align with survey findings related to lower patient volumes in dental practices. During the week of May 18, only 37.6% of dental practices reported patient volumes above 50% of typical patient volume (ADA Health Policy Institute 2020). These numbers offer a glimpse into lost revenue for dental practices in the United States, where more than half of dental practices are solo practices (ADA Health Policy Institute 2018b). Although there are federal loan options available for dental practices from the Small Business Administration, including a Paycheck Protection Program (PPP) loan and Economic Injury Disaster Loan (ADA 2020a), the receipt of these loans among dental practices remains uncertain. For example, 1 news story documented the failure of a dental practice in Chicago to obtain a PPP loan in April 2020 (Sargent 2020). An analysis of PPP loans of $150,000 and $10 million indicated that doctors and dentists borrowed more than $10 billion, yet the percentage obtained by dental practices was not reported (Benincasa 2020). Federal funding is also available from the US Department of Health and Human Services via the Provider Relief Fund for eligible providers who participate in Medicaid and the Children’s Health Insurance Program (ADA 2020b), but this funding may not be available to most dentists because fewer than half of dentists participate in these public insurance programs (ADA Health Policy Institute 2018a).

More than half (52%) of adults reported that they planned to visit the dentist within the next 3 mo, which could be good news for financially struggling dental practices. Whether or not these dental visits occur will likely depend on rates of COVID-19 overall and within a community, patient need and tolerance for risk, and dental providers’ preparedness and tolerance for risk. Dental practices are challenged with how to more safely deliver care, recognizing that usual dental procedures generate aerosols and droplets and that society still has incomplete information about risks of COVID-19 transmission. Guidance for safer dental practice during the pandemic has been issued and updated by both the ADA and CDC (ADA 2020c; CDC 2020b), recommending screening patients for COVID-19 before visits and upon arrival, as well as having providers wear appropriate personal protection equipment (PPE), which may include N95 respirators during aerosol-generating procedures and the highest level of surgical mask, eye protection, face shields, and protective clothing at all times. CDC also recommends avoiding aerosol-generating procedures whenever possible. Both organizations describe their guidance as interim, noting that it will be updated as society learns more about COVID-19. These guidelines illustrate that the reopening of dental practices requires both financial and institutional investment in PPE and new procedures.

Fewer than 1% of survey respondents indicated that they had a virtual dental appointment via the Internet, phone, or video. These virtual dental visits, which can be considered teledentistry, can be used as a tool to triage and screen patients to determine if an in-person visit is necessary. While 22 states had promoted use of teledentistry as of May 7, 2020 (Manatt, Phelps, & Phillips LLP. 2020), dental insurers vary in whether or not they cover teledentistry and the types of services covered (ADA 2020d). Given this variation of teledentistry coverage across and within states, future research should examine its impact on access to dental care.

This study should be interpreted in the context of its limitations. Respondents reported on dental care delayed or forgone due to the COVID-19 pandemic, but we do not know if care was delayed due to patient perceptions of risk, office closures, or other reasons. We also do not know the specific timing of the delayed visit, only that it was before this survey was fielded. Dental practices had little guidance on how to safely deliver care during March 2020, and dental offices in many states were mandated or encouraged to provide only urgent care during March and April 2020 (ADA Center for Professional Success 2020). Survey respondents and nonrespondents exhibited some differences across demographic characteristics, with nonrespondents more likely to be female, younger than 40 y of age, Black, and Hispanic. Furthermore, we cannot be sure if respondents and nonrespondents have similar dental knowledge, behaviors, and preferences. If nonrespondents were less likely to be routine users of dental care, this may lead us to find higher rates of delayed dental care. In addition, if respondents were influenced by social desirability bias, they may have been more likely to respond as if they are routine users of dental care. This may lead us to find higher rates of reported delays of dental care and rates of future use of dental care. In the United States in 2017, 64% of adults reported visiting the dentist in the last year (CDC 2018), suggesting our finding that about half of respondents reported delaying dental care due to the pandemic may potentially be higher than the actual rate. However, as previously noted, widespread closures of dental offices likely made care unavailable to many. This is a cross-sectional study and should not be viewed as making causal claims about the relationships between COVID-19, utilization, and the covariates examined. Importantly, rural and urban communities are heterogenous, but we were unable to explore this variation in our article. Future studies should consider more granular measures of rurality, as well as how availability of dental providers and distance to care may affect delayed care. Finally, this survey was designed to be generalizable to adults in the United States. While it achieved a relatively high response rate of 70%, small samples within some populations limited subanalyses of interest, including examining the experiences of racial and ethnic groups within rural and urban areas.

This study provides important information about US adults’ receipt of dental during the COVID-19 pandemic in the spring of 2020. Our analysis of a nationally representative survey of US adults found that almost half of adults delayed dental care due to the pandemic. This finding has important implications for patient care and dental practice. As the pandemic intensifies across the United States, it remains unclear if adults will return to dental offices and what that will mean for oral health and for the finances of dental practices. While we found that most adults who delayed dental care delayed routine visits, efforts should be made to provide safe and timely care to patients who need dental treatment.

## Author Contributions

A.M. Kranz, contributed to conception, design, data analysis, and interpretation, drafted and critically revised the manuscript; G. Gahlon, contributed to data analysis, drafted the manuscript; A.W. Dick, B.D. Stein, contributed to design and data interpretation, critically revised the manuscript. All authors gave final approval and agree to be accountable for all aspects of the work.

## Supplemental Material

DS_10.1177_2380084420962778 – Supplemental material for Characteristics of US Adults Delaying Dental Care Due to the COVID-19 Pandemic Click here for additional data file. Supplemental material, DS_10.1177_2380084420962778 for Characteristics of US Adults Delaying Dental Care Due to the COVID-19 Pandemic by A.M. Kranz, G. Gahlon, A.W. Dick and B.D. Stein in JDR Clinical & Translational Research
