# Supplementary material for: Characteristics of US Adults Delaying Dental Care Due to the COVID-19 Pandemic
Source: JDR Clin Trans Res. 2020 Sep 27;6(1):8–14. doi: 10.1177/2380084420962778 (PMC7527908; doi:10.1177/2380084420962778)
Supplement: DS_10.1177_2380084420962778 – Supplemental material for Characteristics of US Adults Delaying Dental Care Due to the COVID-19 Pandemic [file DS_10.1177_2380084420962778.pdf]

Appendix material for "Characteristics of US adults delaying dental care due to the COVID-19 pandemic"

Appendix Table. Characteristics of survey respondents

	Weighted Percentage	Unweighted Count
**All**	100.0	2,387
**Sex**		
Male	48.2	1,024
Female	51.8	1,363
**Age Group**		
<40 years	28.7	286
40-54 years	29.2	566
55-64 years	18.8	665
65 years and older	23.4	871
**Race**		
White	75.3	1,920
Black	12.2	221
American Indian and Alaskan Native	1.8	27
Asian and Pacific Islander	3.3	71
Other	7.4	148
**Hispanic ethnicity**		
Hispanic	18.7	334
Not Hispanic	81.3	2,054
**Rurality***		
Urban	78.5	1,860
Rural	21.6	521
**Census division of current residence****		
New England	4.3	111
Middle Atlantic	15.6	321
East North Central	12.9	312
West North Central	4.8	123
South Atlantic	16.6	399
East South Central	3.5	93
West South Central	15.3	343
Mountain	9.9	258
Pacific	17.1	426

Note. Table presents weighted percentages. *Six respondents had missing information about rurality. **One respondent had missing information about Census division.
